# Intrabronchial Foreign Body

**Published:** 1918-07

**Authors:** 


### ABSTRACTS

---

**Intrabronchial Foreign Body.** D. Denechau, Le Prog. Med., Dec. 29, 1917, reports the case of a woman who accidentally swallowed a bit of celluloid from a collar stiffener. Dyspnoea, cough and signs of bronchitis developed, the picture becoming that of pulmonary tuberculosis with emphysema. The foreign body was finally spontaneously ejected in a coughing fit 2 years less three days from the time it was swallowed. Comby, in the discussion, mentioned an analogous case in which the foreign body was not coughed up for more than 20 years.

